# Endoscopic Myringoplasty for Pediatric Tympanic Membrane Perforations: Is It Worth It?

**DOI:** 10.3390/children12030293

**Published:** 2025-02-27

**Authors:** Riccardo Nocini, Daniele Monzani, Valerio Arietti, Flavia Bonasera, Luca Bianconi, Luca Sacchetto

**Affiliations:** Azienda Ospedaliera Universitaria Integrata Verona, Piazzale Aristide Stefani, 1, 37126 Verona, Italy; riccardo.nocini@aovr.veneto.it (R.N.); daniele.monzani@univr.it (D.M.); flavia.bonasera@studenti.univr.it (F.B.); luca.bianconi@aovr.veneto.it (L.B.); luca.sacchetto@univr.it (L.S.)

**Keywords:** myringoplasty, tympanoplasty, tympanic membrane perforation

## Abstract

**Background/Objectives**: The endoscopic repair of the tympanic membrane is an established method for addressing perforations in the tympanic membrane. However, there are limited studies in the literature examining the success rate of this procedure in the pediatric population. **Methods**: This study retrospectively analyzed data from the tertiary referral center at the University Hospital of Verona, Italy. This medical record contains data from 84 type 1 transcanal endoscopic tympanoplasties performed in pediatric patients between November 2014 and February 2022. Seventy-seven pediatric patients aged 4 to 16 years who underwent type 1 transcanal endoscopic tympanoplasty (seven of whom underwent bilateral surgery at different time points) were included in the study. Our study did not include more extensive procedures than type 1 endoscopic tympanoplasty. Only patients with tympanic membrane perforation due to simple chronic otitis media, trauma or when no apparent cause was found were included. Chronic otitis with cholesteatoma and other pathologies of the external or middle ear were exclusion criteria. Patients with a follow-up of less than 12 months were excluded from this study. The technique was based on the endoscopic placement of an underlay graft of temporal fascia or tragal cartilage to repair a tympanic membrane perforation. Demographic, clinical, audiologic, and surgical data were collected from each patient. In the study, we considered the reduction of the air-bone gap (ABG) as a functional outcome and the integrity of the reconstruction as an anatomic outcome of success. **Results**: The primary surgery had a closure rate of 92.9% (78 of 84). All patients underwent audiological evaluation 4–6 months post-surgery, with 84 ears tested. The mean preoperative ABG was 17.13 dB HL, reduced to 9.16 dB HL postoperatively, showing a mean reduction of 7.97 dB HL. No significant complications occurred. **Conclusions**: Transcanal endoscopic type 1 tympanoplasty should be considered a safe procedure with a high success rate for the repair of tympanic membrane perforations, even in pediatric patients.

## 1. Introduction

Since the introduction of ear surgery, tympanic membrane repair has traditionally been achieved by microscopically assisted surgery [1]. In the last decade, the endoscope has become an alternative tool in treating otologic pathologies, and endoscopic ear surgery has taken its place as one of the techniques of choice in treating middle ear cholesteatoma. Currently, type I endoscopic tympanoplasty is widely performed worldwide because of the many advantages of this technique. Perforations of the tympanic membrane are common problems ENT physicians encounter in their practice and are among the most common surgical procedures performed worldwide. This procedure has two main goals:

– full closure of tympanic membrane perforations;
– enhancing hearing level and reducing the air-bone gap (ABG).

In recent years, several studies have shown that the endoscopic technique is as effective or even more effective than the microscopic technique in closing tympanic membrane perforations. Marchioni et al., in a previous article [1], demonstrated an 86% closure rate in a case series of 98 patients, with improvement in ABG within 20 dB HL in 89% of patients. However, most of these studies were performed in adults [1,2,3,4,5,6,7,8,9,10,11,12,13,14]. There is a lack of studies in the literature examining the effectiveness of this procedure in children. One of the most important studies we found was by Kaya et al. [15], in which type I tympanoplasty was performed on both ears with different techniques (microscopic on one side and endoscopic on the other), and the endoscopic technique showed similar results and even better results in terms of pain management and operation time. Many studies have shown controversial results regarding age. Friedberg et al. [16] suggest that the proper age range for performing tympanoplasty is between 7 and 11 years, as they did not record higher graft failure rates in this range. These results may be due to the complete maturation of the eustachian tube at this age. This study describes the endoscopic procedure used at our institution to treat tympanic membrane perforations in children, regardless of size or location. The present study is currently one of the most comprehensive published case series in the pediatric population because the technique was introduced relatively recently. Additional studies with a larger patient cohort are required to validate the findings presented in this paper.

## 2. Materials and Methods

A retrospective cohort study was conducted on 77 pediatric patients, aged 4 to 16 years, who underwent type I endoscopic tympanoplasty between November 2014 and February 2022. This study took place in the Department of Otorhinolaryngology and Head and Neck Surgery at the University Hospital of Verona. The following standardized surgical procedure was employed to treat consecutive patients with tympanic membrane perforation, and it was executed by surgeons in the ENT Department at the University Hospital of Verona. Patients who required a more extensive procedure than type I endoscopic tympanoplasty were not included in our study. The study included only patients diagnosed with chronic otitis media with tympanic membrane perforation, post-traumatic tympanic membrane perforation, or idiopathic perforations. Those who were found to have cholesteatoma preoperatively or intraoperatively were excluded. All patients had perforations involving more than one quadrant. This study did not include patients with minor perforations who underwent fat graft myringoplasty. The follow-up period was at least 12 months; patients whose follow-up time was shorter were excluded from the study. Each patient underwent an endoscopic examination of the ear before surgery. Data on perforation location and size were categorized into anterior, posterior, inferior, and subtotal areas. The data analyzed regarding the surgery included graft types (tragus or concha cartilage, temporalis fascia, bovine pericardium collagen acellular matrices), intraoperative ossicular chain abnormalities, and complications (intraoperative and postoperative). Subsequent evaluations involved endoscopic examinations of the external auditory canal and tympanic membrane at one month and six months post-surgery, with data gathered on tympanic membrane healing, infection rates, reperforation occurrences, and the need for revision surgeries. Audiometric evaluations were conducted to assess hearing function before surgery and 4 to 6 months postoperatively. Hearing outcomes were measured by comparing pre and postoperative air and bone pure tone averages (PTA) at frequencies of 0.5, 1, 2, and 3 kHz. For each patient, the PTA score and air-bone gap (ABG) were calculated both pre and postoperatively. All procedures were carried out by three experienced surgeons, each with a minimum of 8 years of expertise in endoscopic ear surgery.

### 2.1. Preoperative Considerations

Prior to planning an endoscopic transcanal ear exclusive approach, it is essential to consider the patient’s age, the condition of the perforation and tympanic cavity, as well as any previous surgeries for tympanic membrane perforation.

#### 2.1.1. Age

Although age is not an absolute contraindication for endoscopic ear surgery, special caution should be exercised when performing type I endoscopic tympanoplasty on children under six years of age. This precaution is necessary due to the incomplete maturation of the rhinotympanic system and the relatively small size of the external auditory canal (EAC). Children have a higher likelihood of developing infections that can lead to a new perforation and potential failure of the surgery. Nevertheless, the straight shape of the ear canal in children provides a favorable condition for performing endoscopic ear surgery.

#### 2.1.2. Perforation and Condition of the Tympanic Cavity

Type I endoscopic tympanoplasty was not conducted if unstable conditions were present in the tympanic cavity, such as infection or recurrent otorrhea, because these conditions could adversely affect the closure rate.

#### 2.1.3. Graft Selection

Suitable grafting materials include cartilage, temporalis fascia, vein grafts, fascia lata, and perichondrium. Temporalis fascia is the most commonly used and has a high success rate for closures. Cartilage grafts have recently been increasingly used, especially in complex perforations or revision procedures. In our study, the graft material was selected similarly to what we chose for adults (as shown in Table 1), using cartilage grafts for complex cases, such as anterior or more than two quadrant perforations [17]. Xenografts are tissues from non-human species that have been engineered for use as graft material in type I tympanoplasty, intended for tympanic membrane repair similarly to temporalis fascia. Acellular collagen matrices derived from bovine pericardium (Tutopatch—Tutogen Medical Inc., Alachua, FL, USA) are utilized by our department.

#### 2.1.4. Compliance with Ethical Standards

This study complied with the ethical standards of the institutional and national research committees, as well as the 1964 Declaration of Helsinki and its amendments. Informed consent was obtained from all participants.

#### 2.1.5. Surgical Procedure

##### Position of the Patient

The patient’s positioning remains consistent with that employed for microscopic surgery. The skin of the auricular region and external auditory canal is prepared using a povidone-iodine solution. The primary surgeon utilizes the endoscope with their non-dominant hand while manipulating surgical instruments or suction with their dominant hand. The endoscopes utilized included a 0°, 3-mm-width, 14-cm-length endoscope (Karl Storz, Tuttlingen, Germany) or a 0°, 4-mm-width, 18-cm-length endoscope. In cases where middle ear pathology, particularly middle ear cholesteatoma, was suspected, a rigid 45° endoscope (45°, 14 cm, 3 or 4 mm wide) was used to examine the anatomy of the middle ear and concealed areas in greater detail. The tympanomeatal flap design is tailored to the defect’s location and size.

##### Surgical Steps

Most of the procedures were performed utilizing a 0°, 3-mm, 14-cm endoscope. Initially, the external auditory canal (EAC) is infiltrated with a solution of 2% mepivacaine and epinephrine, administered 3 min before the procedure. To facilitate graft integration, scarring of the perforation margins is performed using an otologic sickle knife. An incision is created using an otologic round knife in the external auditory canal (EAC) skin, approximately 5–10 mm from the annulus, positioned between the 6 and 12 o’clock marks. The primary incision can be initiated with a monopolar scalpel to create a dotted line for coagulation and reduced bleeding, and then completed with a round knife, based on the surgeon’s preference. The tympanomeatal flap is carefully detached from the EAC bone and elevated until the annulus is visible, using the round knife and a cotton ball soaked in epinephrine to protect the tympanic membrane and control bleeding. Any bleeding is promptly managed with epinephrine-soaked cotton balls applied for one minute. The mucosa of the middle ear is then incised, and the fibrous annulus is elevated using a cotton ball while preserving the integrity of the chorda tympani. The flap is lifted from posterior to anterior, remaining attached to the malleus handle. Complete visualization of the middle ear cavity is achieved after detaching the posterior malleolar ligament. In the case of posterior perforations, it may not be required to completely detach the tympanic membrane from the malleus. Adequate management of the anterior margin of this type of perforation can still be achieved. In cases of anterior perforations, the periosteum is detached from the handle of the malleus using a sickle knife. The flap is then separated from the ossicular chain at the subperiosteal level while leaving the umbo attached. Micro-scissors are used to separate at the umbo. The membrane is moved forward while staying connected to the anterosuperior wall of the external auditory canal. The mobility of the ossicular chain is then assessed. A thorough examination of the entire tympanic cavity is conducted using angled endoscopes to rule out middle ear pathology (e.g., cholesteatoma) and detect possible isthmus obstruction, which can affect middle ear ventilation and subsequent procedural success. The chosen graft is carefully shaped to match the size of the perforation and is then inserted laterally on the malleus handle using a sickle knife, ensuring that the edges remain beneath the fibrous annulus (underlay technique). In instances of posterior perforation, the graft can be positioned medial to the malleus handle without totally detaching the flap. When utilizing temporalis fascia as the graft material, it is crucial to compress and adequately dry it to enhance its stiffness and maneuverability, which is particularly essential for sliding the graft under the anterior segment of the tympanic tube and the fibrous ring. For cartilage grafts, the perichondrium is left intact on one side, with approximately 1 mm overhanging the posterior edge of the cartilage and removed from the other side. The extracted perichondrium is preserved in case another graft becomes necessary, with the perichondrium being pressed and dried similar to the fascia. Once the graft is precisely shaped, an incision is crafted to accommodate the malleus handle and the posterior sulcus. The cartilage is then inserted, ensuring that the perichondrium side contacts the tympanomeatal flap, and the perichondrium of the posterior edge of the cartilage graft aligns with the posterior wall of the external auditory canal (EAC). If temporal fascia is used, the graft is elevated anterosuperiorly and secured to the tympanomeatal flap. Gel foam is introduced into the tympanic cavity to serve as a scaffold for the graft, with care taken to prevent obstruction of the eustachian tube or isthmus by scar tissue. The graft and tympanomeatal flap are meticulously repositioned. The endoscopic view provides excellent visualization of the anterior edges of the annulus fibrosus ring and its position relative to the graft, ensuring optimal outcomes. Small quadrangular pieces of Gelfoam are positioned at the edges of the perforation and flap incision to assist in healing, protect the graft, and keep it in place. Subsequently, the external ear canal is filled with Gelfoam pieces, ensuring no pressure is applied to the tympanic membrane.

#### 2.1.6. Postoperative Care and Follow-Up

If no complications occur, patients are discharged the next day. They should avoid actions that increase middle ear pressure, such as Valsalva maneuvers, sneezing, abdominal contractions, and nose blowing. Intense exercise and high-altitude flying are discouraged. Patients are advised to avoid getting water in the operated ear and to apply antibiotic ear drops twice daily for a month to prevent infections. A follow-up endo-otoscopic exam is conducted one month post-surgery to confirm healing before resuming normal activities. Audiometry tests are performed four months after surgery to assess results. Annual endo-otoscopy is recommended for at least two years. After discharge, it is crucial for patients to adhere strictly to post-operative instructions to ensure optimal healing and to prevent complications. Any signs of infection, such as fever, increased pain, or unusual discharge from the ear, should be reported to the healthcare provider immediately. While using antibiotic ear drops, ensure the medicine reaches deep into the ear canal by tilting the head sideways for a few minutes after application. For those who experience discomfort or mild pain, over-the-counter pain medications like acetaminophen or ibuprofen may be used unless contraindicated. It is also advisable to avoid environments with extreme temperatures or humidity, which could potentially affect the ear’s healing process.

## 3. Results

Seventy-seven pediatric patients were included in our study, for a total of 84 procedures (seven bilateral procedures) completed between November 2014 and February 2022 at the Department of Otolaryngology, Head and Neck Surgery of the University Hospital of Verona. Of the 77 patients selected, 12 had a bilateral perforation, but only 7 underwent bilateral surgery, bringing the number of type I tympanoplasty procedures to 84. Regarding laterality, 42 right (50%) and 42 (50%) left perforations were treated. The male-to-female ratio was 49:35. The mean age at surgery was 10.2 years (range 4–16 years). Perforations were categorized according to size and location and were divided into the following categories: anterior (30 perforations, 35.7%), inferior (23 perforations, 27.4%), posterior (9 perforations, 10.7%) and subtotal (22 perforations, 26.2%). In all patients, type I endoscopic tympanoplasty was performed using only the abovementioned technique. The graft material chosen was mainly autologous, with tragal cartilage in 34 cases (40.5%) and autologous temporalis muscle fascia in 43 cases (51.2%) (Table 2). In all cases where only tragal cartilage was utilized, no additional perichondrium was necessary, as the provided cartilage was sufficient. In our study, two complications were observed, one intraoperatively (chorda tympani section) and one postoperatively (iatrogenic cholesteatoma limited to mesotympanum which needed a revision surgery through an endoscopic exclusive transcanal approach). Endoscopic examinations were performed in all 77 patients (84 surgeries in total) 1, 6 and 12 months after surgery. Persistent perforation after surgery occurred in only six (7.14%) patients 1 month after surgery. Among the failures, one occurred in a patient with preoperative subtotal perforation (1 out of 22, or 4.5%), and five occurred in patients with preoperative anterior perforation (5 out of 30, or 16.67%). Three failures were observed in patients with chronic otitis perforations, two failures in cases of idiopathic perforation, and one failure resulted from trauma. Thus, the closure rate of primary surgery was 92.85% (78 of 84). In all failed operations, the graft material of choice was temporalis muscle fascia. The average duration of the surgical procedure (the time from injection of local anesthesia to closure of the ear canal) was 69.5 (34–132) minutes for all 84 procedures in which endoscopic transcanal tympanoplasty type I was performed.

All patients were audiometrically assessed between 4 and 6 months postoperatively, and a total of 84 ears were tested. The mean preoperative ABG value was 17.13 dB HL, whereas the mean postoperative ABG value was 9.16 dB HL, corresponding to a mean ABG reduction of 7.97 dB HL. An ABG value of less than 20 dB HL was considered an appropriate audiological outcome. This was achieved in 91.7% (77 of 84) of cases, with 31% (26 of 84) of cases displaying a hearing improvement of at least 10 dB HL (in terms of ABG closure). Hearing performance worsened slightly in only 3.6% (3 of 84) of cases; no worsening > 10 dB HL was noted. It is important to note that there was no deterioration of bone conduction, which means that the interventions caused no sensorineural hearing loss. There were no changes in ABGs in 4.8% (4 of 84) of cases.

## 4. Discussion

Minimally invasive surgery is being adopted more frequently in various surgical disciplines. In the field of ENT surgery, the endoscope has been effectively integrated into otologic approaches. Traditionally, middle ear surgeries performed under microscopic vision are now frequently conducted using endoscopic techniques. The microscopic approach allows the surgeon to operate with both hands, which aids in tissue manipulation and bleeding control. On the other hand, the microscopic transcanal approach does not allow for complete exposure of the tympanic membrane and its medial surface, hindering visualization of the anterior margin of perforations. Conversely, the endoscope offers precise visualization of these areas. It offers a clear view of the tympanic membrane, especially in patients with intricate external ear anatomy, such as narrow and curved ear canals. The enhanced visibility afforded by the endoscope enables reliable refreshment of the perforation margins and successful grafting [18]. Furthermore, this technique typically obviates the need for canaloplasty even in patients with narrow or tortuous ear canals. It also reduces postoperative bleeding and pain, and yields superior cosmetic results compared to the traditional microscopic retro auricular approach, while minimizing local and general postoperative side effects. The transcanal endoscopic technique does not require cutting or damaging the vascular strip that supplies blood from the malleolar and radial arteries to the tympanic membrane, thereby facilitating faster wound healing [19]. Since 2014, our center has refined the endoscopic type I tympanoplasty technique for rapid flap formation, good bleeding control, and easy pad grafting. This study shows the feasibility and benefits of the transcanal endoscopic method in children. The technique successfully repaired the tympanic membrane in all patients by treating the perforation margins. It can be performed regardless of perforation size or location, even with narrow or tortuous ear canals. In this series, no procedures required switching to the traditional microscopic technique. Among 77 patients and 84 procedures, the primary surgery closure rate was 92.85%. The literature reports overall closure rates between 84% and 96%, consistent with our findings [4,20,21]. The success rate for closure in type I tympanoplasty using microscopic techniques has historically ranged between 78% and 90% [21,22]. Improving hearing is crucial for enhancing future quality of life. Numerous studies have documented positive outcomes in postoperative hearing gain for patients who have undergone endoscopic type I tympanoplasty. Lade et al. [17] reported a mean ABG closure in pediatric patients undergoing type I endoscopic tympanoplasty of 10.37 dB versus a mean ABG closure of 15.5 with the microscopic technique, without statistical significance. Our results were similar to those observed in the literature. The mean preoperative ABG value was 17.13 dB HL, whereas the mean postoperative ABG value was 9.16 dB HL, corresponding to a mean ABG reduction of 7.97 dB HL. An ABG value of less than 20 dB HL was considered an appropriate audiological outcome. This was achieved in 91.7% (77 of 84) of cases. Hearing performance worsened slightly in only 3.6% (3 of 84) of cases; no worsening > 10 dB HL was observed. This means that type 1 endoscopic tympanoplasty has a very high success rate in closing tympanic membrane perforations and guarantees an excellent audiological outcome. A key benefit of endoscopic procedures in middle ear surgery is the reduction in surgical duration, which is significant for minimizing anesthesia time. This advantage may be attributed to the fact that endoscopic techniques do not necessitate sutures and allow for faster visualization of concealed areas. In our study, the mean duration of the surgical procedure was 69.5 min, with a range of 30 to 145 min. Some researchers suggest that the lateral placement of the fascia or cartilage graft on the malleus handle may pose a risk for delayed blunting of the neo-tympanum [23]. The over–under technique described by Kartush et al. [24], where the graft is positioned lateral to the malleus handle and medial to the tympanic ring, has shown effective functional results and closure rates in type I microscopic tympanoplasty. In this study, no cases of blunting were noted at follow-up, indicating the practicality of this technique. It highlights that precise placement of the graft under the annulus in its anterior region is crucial to prevent lateral displacement. Though the number of cases is too small for statistical analysis, we observe that five out of six surgery failures with reperforation were anterior, using temporalis muscle fascia as the graft. This may suggest that temporalis muscle fascia is not preferred when the perforation is anterior in children. Anterior perforations present a greater challenge to manage using both endoscopic and microscopic approaches. This may be considered a limitation of the technique. However, it is important to note that anterior perforations treated with cartilage have been successfully managed. Further research is required to validate these findings. This could be a valid topic of debate, especially considering that new techniques are emerging, such as tympanic membrane regeneration therapy used to treat tympanic membrane perforation (TMP) based on a tissue engineering method developed by Kanemaru et al. [25] In this treatment, the perforated TM is mechanically freshly wounded through the external auditory canal, and a gelatin sponge impregnated with trafermin (recombinant human basic fibroblast growth factor) is placed inside and outside the TM so that it contacts the perforated TM. This minimally invasive technique requires no skin incision or autologous tissue collection.

The present study is currently one of the most comprehensive published case series in the pediatric population because the technique was introduced relatively recently. Further studies with more patients are needed to confirm the data presented in this paper.

This study is retrospective and, therefore, has some limitations. One of the most important limitations is that there might be a bias in the selection of patients because the choice of the graft was based on our experience and did not follow the rules of randomization. Another limitation is the lack of microscopic control in type I tympanoplasty, even though microscopes are widely used and effective for other procedures such as stapedoplasty [26]. Thus, we had to rely on the general literature, which is often variable. We tried to exclude any obvious bias because this was a consecutive series of patients with strict inclusion and exclusion criteria, treated in the same way and with the same technique by three experienced surgeons at the exact center. In any case, this is one of the studies in the literature with the highest number of pediatric patients treated with EES for repair of MT.

## 5. Conclusions

Endoscopic ear surgery has now become a viable alternative to traditional microscopic ear surgery in many cases. Our center has one of the largest caseloads globally. While this study does not claim that the endoscopic technique is superior to the microscopic one, it demonstrates that type I transcanal endoscopic tympanoplasty can be considered a safe and effective technique with a high success rate for treating tympanic membrane perforations, even in young patients.

## Figures and Tables

**Table 1:** Choice of graft material.

Graft Material	Indications
Temporalis fascia	Perforation confined to one sector of the tympanic membrane (better for posterior perforations)
Tragal cartilage	Wide perforations with more than one affected sector or involvement of the annulus or failure of the fascia temporalis
Conchal cartilage	Earlier failures when no tragal cartilage is available
Xenograft	Never used as first choice, sometimes used to reinforce reconstruction

**Table 2:** Main results.

	n	%
TM Perforations	84	100.0
Side		
Left	42	50.0
Right	42	50.0
Gender		0.0
Female	35	41.7
Male	49	58.3
Mean age (years)	10.2	(4–16)
Perforation location		
Anterior	30	35.7
Posterior	9	10.7
Inferior	23	27.4
Subtotal	22	26.2
Graft		
Temporalis muscle fascia	34	40.5
Tragal cartilage	43	51.2
Temporalis muscle fascia + xenograft	3	3.6
Tragal cartilage + xenograft	2	2.4
Temporalis muscle fascia + tragal cartilage	1	1.2
Xenograft	1	1.2
Complications		
None	82	97.6
Chorda tympani damaged	1	1.2
Cholesteatoma	1	1.2
Surgery duration (minutes)	69.5	(34–132)
TM repair		
Yes	78	92.9
No	6	7.1
Initial perforation location in failure		
Inferior	5	6.0
Subtotal	1	1.1
Graft used in failure		
Temporalis muscle fascia	5	6.0
Temporalis muscle fascia + xenograft	1	1.1
BC PTA (mean)		range
Preoperative	9.24	(5–26.25)
Postoperative	8.61	(5–26.25)
AC PTA (mean)		
Preoperative	26.69	(10–48.75)
Postoperative	17.77	(10–37.5)
ABG (mean)		
Preoperative	17.13	(5–33.75)
Postoperative	9.16	(10–26.75)
Ears with normal hearing (AC PTA < 20 dB HL)		
Preoperative	29 of 84	34.5
Postoperative	62 of 84	73.8
Pre vs post ABG		
>10 improvement	26	31.0
0–10 improvement	51	60.7
no change	4	4.8
0–10 worsening	3	3.6
>10 worsening	0	0.0

## Data Availability

The original contributions presented in this study are included in the article. Further inquiries can be directed to the corresponding author.

## References

[1] Marchioni D., Gazzini L., De Rossi S., Di Maro F., Sacchetto L., Carner M., Bianconi L. (2020). The Management of Tympanic Membrane Perforation With Endoscopic Type I Tympanoplasty. Otol. Neurotol..

[2] Presutti L., Marchioni D. (2015). Endoscopic Ear Surgery Principles, Indications, and Techniques.

[3] Özdemir D., Özgür A., Akgül G., Çelebi M., Mehel D.M., Aydemir S., Yemiş T. (2019). Transcanal Endoscopic Type 1 Cartilage Tympanoplasty in Children. Turk. Arch. Otorhinolaryngol..

[4] Lakpathi G., Sudarshan Reddy L., Anand (2016). Comparative Study of Endoscope Assisted Myringoplasty and Microscopic Myringoplasty. Indian J. Otolaryngol. Head Neck Surg..

[5] Jyothi A.C., Shrikrishna B.H., Kulkarni N.H., Kumar A. (2017). Endoscopic Myringoplasty Versus Microscopic Myringoplasty in Tubotympanic CSOM: A Comparative Study of 120 Cases. Indian J. Otolaryngol. Head Neck Surg..

[6] Yadav S.P., Aggarwal N., Julaha M., Goel A. (2009). Endoscope-assisted myringoplasty. Singapore Med. J..

[7] Usami S., Iijima N., Fujita S., Takumi Y. (2001). Endoscopic-assisted myringoplasty. ORL J. Otorhinolaryngol. Relat. Spec..

[8] Raj A., Meher R. (2001). Endoscopic transcanal myringoplasty-A study. Indian J. Otolaryngol. Head Neck Surg..

[9] Tarabichi M. (1999). Endoscopic middle ear surgery. Ann. Otol. Rhinol. Laryngol..

[10] Harugop A.S., Mudhol R.S., Godhi R.A. (2008). A comparative study of endoscope assisted myringoplasty and micrsoscope assisted myringoplasty. Indian J. Otolaryngol. Head Neck Surg..

[11] Lee S., Cho H.H. (2020). Transcanal Endoscopic Tympanoplasty for Pediatric Patients Under 15 Years of Age with Chronic Otitis Media. Clin. Exp. Otorhinolaryngol..

[12] Dündar R., Kulduk E., Soy F.K., Aslan M., Hanci D., Muluk N.B., Cingi C. (2014). Endoscopic versus microscopic approach to type 1 tympanoplasty in children. Int. J. Pediatr. Otorhinolaryngol..

[13] Coskun B.U., Cinar U., Seven H., Ugur S., Dadas B. (2006). The effects of the incision types in myringoplasty operations on cosmesis. Eur. Arch. Otorhinolaryngol..

[14] Sen A., Özdamar K. (2019). Which graft should be used for the pediatric transcanal endoscopic type 1 tympanoplasty? A comparative clinical study. Int. J. Pediatr. Otorhinolaryngol..

[15] Kaya I., Sezgin B., Sergin D., Ozturk A., Eraslan S., Gode S., Bilgen C., Kirazli T. (2017). Endoscopic versus microscopic type 1 tympanoplasty in the same patients: A prospective randomized controlled trial. Eur. Arch. Otorhinolaryngol..

[16] Friedberg J., Gillis T. (1980). Tympanoplasty in childhood. J. Otolaryngol..

[17] Lade H., Choudhary S.R., Vashishth A. (2014). Endoscopic vs microscopic myringoplasty: A different perspective. Eur. Arch. Otorhinolaryngol..

[18] Lou Z.C. (2020). Endoscopic myringoplasty: Comparison of double layer cartilage-perichondrium graft and single fascia grafting. J. Otolaryngol. Head Neck Surg..

[19] Marchioni D., Rubini A., Gazzini L., Alicandri-Ciufelli M., Molinari G., Reale M., Presutti L. (2018). Complications in Endoscopic Ear Surgery. Otol. Neurotol..

[20] Pap I., Tóth I., Gede N., Hegyi P., Szakács Z., Koukkoullis A., Révész P., Harmat K., Németh A., Lujber L. (2019). Endoscopic type I tympanoplasty is as effective as microscopic type I tympanoplasty but less invasive-A meta-analysis. Clin. Otolaryngol..

[21] Ryan M.A., Kaylie D.M. (2016). What is the optimal age to repair tympanic membrane perforations in pediatric patients?. Laryngoscope.

[22] Nicholas Jungbauer W., Jeong S., Nguyen S.A., Lambert P.R. (2023). Comparing Myringoplasty to Type I Tympanoplasty in Tympanic Membrane Repair: A Systematic Review and Meta-analysis. Otolaryngol. Head Neck Surg..

[23] Vartiainen E., Kärjä J., Karjalainen S., Härmä R. (1985). Failures in myringoplasty. Arch. Otorhinolaryngol..

[24] Kartush J.M., Michaelides E.M., Becvarovski Z., LaRouere M.J. (2002). Over-under tympanoplasty. Laryngoscope.

[25] Kanemaru S., Kita S., Kanai R., Yamaguchi T., Kumazawa A., Yuki R., Yoshida M., Miwa T., Harada H., Maetani T. (2024). Tympanic Membrane Regeneration Therapy for Pediatric Tympanic Membrane Perforation. Otol. Neurotol..

[26] Sacchetto L., Raguso G., Confuorto G., Arietti V., Torroni L., Marchioni D., Nocini R. (2024). A comparison between endoscopic and microscopic approaches for stapes surgery: Experience of a tertiary referral center. Eur. Arch. Otorhinolaryngol..

